# Identification of supraoptimal temperatures in juvenile blueback herring (*Alosa aestivalis*) using survival, growth rate and scaled energy reserves

**DOI:** 10.1093/conphys/coac022

**Published:** 2022-04-20

**Authors:** Lian W Guo, Adrian Jordaan, Eric T Schultz, Stephen D McCormick

**Affiliations:** Program in Organismic and Evolutionary Biology, University of Massachusetts, 230 Stockbridge Road, Amherst, MA 01003, USA; Department of Environmental Conservation, University of Massachusetts, 160 Holdsworth Way, Amherst, MA 01003, USA; Department of Ecology and Evolutionary Biology, University of Connecticut, 69 N Eagleville Rd, Storrs, CT 06269, USA; Department of Environmental Conservation, University of Massachusetts, 160 Holdsworth Way, Amherst, MA 01003, USA; US Geological Survey, Eastern Ecological Science Centre, S.O. Conte Research Laboratory, 1 Migratory Way, Turners Falls, MA 01376, USA

## Abstract

For young fishes, growth of somatic tissues and energy reserves are critical steps for survival and progressing to subsequent life stages. When thermal regimes become supraoptimal, routine metabolic rates increase and leave less energy for young fish to maintain fitness-based activities and, in the case of anadromous fishes, less energy to prepare for emigration to coastal habitats. Thus, understanding how energy allocation strategies are affected by thermal regimes in young anadromous fish will help to inform climate-ready management of vulnerable species and their habitat. Blueback herring (*Alosa aestivalis*) are an anadromous fish species that remain at historically low population levels and are undergoing southern edge-range contraction, possibly due to climate change. We examined the effects of temperature (21°C, 24°C, 27°C, 30°C, 33°C) on survival, growth rate and energy reserves of juveniles collected from the mid-geographic range of the species. We identified a strong negative relationship between temperature and growth rate, resulting in smaller juveniles at high temperatures. We observed reduced survival at both 21°C and 33°C, increased fat and lean mass-at-length at high temperatures, but no difference in energy density. Juveniles were both smaller and contained greater scaled energy reserves at higher temperatures, indicating growth in length is more sensitive to temperature than growth of energy reserves. Currently, mid-geographic range juvenile blueback herring populations may be well suited for local thermal regimes, but continued warming could decrease survival and growth rates. Blueback herring populations may benefit from mitigation actions that maximize juvenile energy resources by increasing the availability of cold refugia and food-rich habitats, as well as reducing other stressors such as hypoxic zones.

## Teaser

We measured anadromous juvenile blueback herring survival, growth rate and energy reserves across temperatures 21–33°C when fed a constant ration. Growth was more sensitive to warm temperatures than energy reserves. Survival remained high, yet smaller size at high temperatures may detrimentally delay emigration, increase predation and lower physiological resilience.

## Introduction

Temperature is a controlling factor of biochemical processes critical to metabolism in all living organisms (Fry, 1947). Thus, body temperature, the intake of energetic resources and the presence of energetically costly environmental factors determine the available energy an organism can utilize to maintain normal physiological functions and activities critical for fitness (Brett and Groves, 1979; Fry, 1947). The influence of temperature and other habitat factors is critical during early life stages of fishes, when young-of-year fish are energy-limited and particularly susceptible to size-based mortality (Sogard, 1997; Weatherley, 1990; Weatherley *et al.*, 1987). Temperature-dependent physiological traits, such as growth rate, are often represented by thermal performance curves, indicating that the trait is maximized at an ‘optimal’ temperature (Schulte *et al.*, 2011); lower and higher temperatures may thus be regarded as suboptimal and supraoptimal, respectively. Characterizing the shape of thermal performance curves has renewed importance in conservation efforts due to climate change. If we understand which temperatures lead to decreased survival, growth and energy reserves in early life stages, then we can inform population-level predictions of climate change impacts and thus improve decision-making for mitigation efforts.

Size, energy reserves and their interaction influence juvenile fish fitness and thus, population sustainability. Low energy content reduces tolerance to starvation and physiological disturbances (i.e. physiological resilience), resulting in increased overwintering mortality (Lee *et al.*, 2015; Schultz and Conover, 1999). For anadromous fishes, emigration from nursery to coastal habitats poses additional energetic demands due to habitat changes that require acclimation, such as salinity, and navigation of migration barriers. The quality of freshwater nursery habitats is highly variable depending on site hydrography, terrestrial inputs and biological assemblages, and significant abiotic and biotic changes are predicted as a result of global climate change (Ficke *et al.*, 2007). Anadromous juvenile fish energy budgets may therefore be increasingly constrained and could provide an early indication of climate effects in aquatic environments. Reduced survival, growth rates or energy reserves due to suboptimal rearing habitat conditions can result in significant delays in emigration timing and reduced emigration success. For instance, both size and energy reserves affect anadromous juvenile emigration timing, as migratory juveniles are larger and in greater body condition than non-migrants (Gahagan *et al.*, 2010; Jonsson and Jonsson, 2002), and faster growth and larger size at emigration in juvenile salmonids yield increased survival to maturity (Thompson and Beauchamp, 2014; Zabel and Williams, 2002).

Blueback herring (*Alosa aestivalis*) are an anadromous clupeid species that was historically distributed along the eastern North American continent, from St. Johns River, FL, USA, to Nova Scotia, Canada. Early life stages of blueback herring are found from late spring to late fall in various freshwater (main stem, tributaries, lakes, ponds) and estuarine habitats (Payne Wynne *et al.*, 2015; Turner and Limburg, 2016). Blueback herring populations remain at historically low levels, likely due to a combination of overfishing, habitat loss, predation and climate change (Davis *et al.*, 2012; Hall *et al.*, 2011; Schmidt *et al.*, 2003). Thus, blueback herring are a target of ongoing restoration efforts because of the critical role they, and the congeneric species alewife (*Alosa pseudoharengus*), hold as an abundant lipid-rich resource for aquatic and terrestrial life in coastal and oceanic environments (Dias *et al.*, 2019; Durbin *et al.*, 1979; Walters *et al.*, 2009). Several studies have identified the relative importance of adult blueback herring population sizes, density dependence and abiotic factors on juvenile abundance (Devine *et al.*, 2021; Kosa and Mather, 2001; Tommasi *et al.*, 2015), but there is little mechanistic understanding of how temperature affects productivity in nursery habitats, especially in the context of climate change projections.

Blueback herring may already be experiencing significant effects of climate change throughout their native range. Field studies demonstrate that in some systems, temperature correlates with juvenile abundance or density, growth rate and emigration timing (Devine *et al.*, 2021; Jessop, 1994; Tommasi *et al.*, 2015). Moreover, retrospective analysis of trawl survey data (1975–2012) tracks a northward shift in the centre of adult blueback herring distribution in both spring and fall seasons (Nye *et al.*, 2012). The effects of reductions in growth rates and energy reserves due to elevated temperature are of significant concern throughout the blueback herring range. In some aquatic species, temperate populations have limited time to benefit from rapid growth in warm and food-rich conditions (Conover, 1992; Schultz and Conover, 1997), while populations near the equator may have limited capacity for adaptation due to narrow thermal safety margins (Hochachka and Somero, 2002; Pinsky *et al.*, 2019). Examining the influence of temperature on survival, growth and energy reserves in early life stages of blueback herring could help elucidate whether northward shifts are limited by juvenile productivity and characterize the suitability of prospective nursery habitats targeted in restoration efforts. We expect that, similar to other anadromous fishes, reduced juvenile growth rates or energy reserves could increase size-selective mortality or delay ontogenetic shifts (e.g. emigration to estuaries) (Gahagan *et al.*, 2010; Iafrate and Oliveira, 2008; Juanes *et al.*, 1993).

Current information on temperature effects on the energetics of anadromous juvenile blueback herring is limited. Previous measures of juvenile growth in relation to temperature use change in size distribution over time as proxies for individual growth and assume that juveniles resampled at field sites are from the same cohort (Burbidge, 1974; Jessop, 1994), or estimate growth through the use of otoliths or growth models rather than directly measure change in size over time (Alexander *et al.*, 2020; Iafrate and Oliveira, 2008; Tuckey, 2009). Existing experimental studies on blueback herring examined larval growth, or measured the effects of salinity rather than temperature (DiMaggio *et al.*, 2015; Schubel *et al.*, 1977; Sismour, 1994). To our knowledge, no previous experimental studies have directly measured growth or energy reserves of juvenile blueback herring in relation to temperature. Therefore, we utilized an experimental approach to test the effects of temperature on anadromous juvenile blueback herring survival, specific growth rate and size-scaled energy reserves when fed a constant ration. We conducted two experiments to assess how temperature effects differ among stages of juvenile development. We chose an experimental thermal range (21°C, 24°C, 27°C, 30°C, 33°C) that represents temperatures typically observed from May to October (i.e. during freshwater residence of blueback herring in the mid- to southern range of their distribution). We predicted that survival, growth rate and energy reserves would decline at the highest temperatures (i.e. would be supraoptimal) and smaller juveniles would be more sensitive to high temperatures. Identifying the thermal limits to juvenile survival, growth and energy reserves may explain current patterns of productivity for blueback herring and improve climate-preparedness for ongoing habitat restoration efforts. This research also demonstrates how accounting for experimental fish size can illuminate consistent as well as divergent patterns in energy allocation within a life stage.

## Methods

### Ethical statement

The care and use of experimental animals complied with US animal welfare laws, guidelines and policies as approved by University of Massachusetts Amherst Institutional Animal Care and Use Committee (research facility #14-R-0036, permit #135). We euthanized all fish at the conclusion of the experiment for tissue sampling.

### Collection and housing

Using collection methods described in Devine *et al.* (2018), we collected juvenile blueback herring via several purse-seine hauls after sunset (2000–2400 hours) from Wethersfield Cove, CT, USA (41.725964, −72.658727), on 2 July 2019 (Experiment 1, E1) and 5 August 2019 (Experiment 2, E2). To reduce transport stress, we added ProLine Aqua-Coat and Instant Ocean Sea Salt (5 ppt) to aerated plastic bags stabilized within round solid plastic containers (diameter, 0.66 m). Juveniles were transported to Cronin Aquatic Resource Center in Sunderland, MA, USA, and transferred to a 949-l stock tank with recirculating natural pond water maintained within 2°C of the collection temperature (E1, 27°C; E2, 25°C). Within 24 hours of collection, we ramped the stock tank temperature 1°C h^−1^ to the target acclimation temperature for each experiment (E1, 21°C; E2, 24°C). This rate of temperature change is ecologically relevant for riverine species in our region (Chadwick *et al.*, 2015), and no acute negative impacts were observed. We acclimated E2 fish to a higher temperature to better characterize the upper thermal growth limit. We trained juveniles on *ad libitum* Otohime commercial grow-out fish feed (80–20% mix of Otohime B2 and C1, 360–840 μm, 51% crude protein, 11% crude fat); 100% of fish fed consistently within 3 days post-collection.

### Experimental design

Once juveniles were trained on dry food (3 days), we haphazardly transferred fish to eight experimental recirculating tanks (116 l) maintained at the acclimation temperature 4–5 days prior to the start of each experiment. Thus, juveniles were maintained in acclimation temperatures for a total of 7–8 days, which has been shown sufficient for stabilized growth rates and survival rates after new temperature exposures in other river herring juveniles (Guo *et al.*, 2021). Juveniles were stocked at a density of 0.22–0.34 fish l^−1^ (*n* = 26–40). Four sump tanks supplied the eight tanks, such that two experimental tanks shared a sump tank. We randomly assigned each set of two tanks one of four temperature treatments, as follows: E1 [21°C, 24°C, 27°C, 30°C] and E2 [24°C, 27°C, 30°C, 33°C].

To calculate growth rate, we weighed fish prior to the experiment (Day −2) and weekly thereafter until they were sampled on experimental Day 21. We used a rapid method to individually weigh juveniles without anaesthesia as described in Guo *et al.* (2021), because blueback herring are sensitive to MS-222 anaesthesia. Briefly, after adding ProLine Aqua-Coat and adjusting tank salinity to 2 ppt to reduce stress, we individually netted fish and placed them in a synthetic chamois-lined 120-ml specimen cup, we measured total mass and then released the fish. Final fish mass accounted for water that adhered to the fish when it was weighed by subtracting cup mass after the fish was removed. It took 20–30 minutes to weigh all individuals in a tank. Thirty minutes after we weighed all fish, incoming water was turned on, causing the salt to equilibrate to 1 ppt within 15 minutes. We performed a 50% water change to bring the salinity back to 0.5 ppt. We found that juveniles on Day −2 were of a similar mass in all experimental tanks both in E1 and E2 (generalized linear model, all *P* > 0.050; Supplementary Table 1), and that E2 fish were approximately five times larger than E1 fish (mean ± SD: E1, 197 ± 80 mg; E2, 1063 ± 262 mg).

The day before the start of the experiment (Day −1), we steadily ramped temperature at an average rate of 1°C h^−1^ until the tank reached the experimental temperature. We initiated the experiments on 10 July 2019 (E1) and 14 August 2019 (E2), when we began feeding 3% daily rations. All masses of fish within a tank were summed to calculate a total tank biomass. The tank biomass (based on previous day) was multiplied by 0.03 (3%) to determine the mass of daily ration for each tank. We weighed an 80–20% mix of Otohime B2 and C1 into two portions, such that the fish were delivered half of their daily ration (1.5% of tank biomass) in each feeding. A 3% ration was chosen to provide fish with plentiful, but controlled food resources; maximum consumption rates estimated for juvenile blueback herring from the same system (our unpublished work) ranged from 2.9% to 3.8% biomass day^−1^ for temperatures 24–32°C. We recorded and measured (total length and mass) daily mortalities and subtracted the mass of dead fish from the tank biomass. All fish were weighed weekly to update the estimated tank biomass for measuring growth rates and adjust the daily ration.

We ended E1 on 31 July 2019 (21 days) and E2 on 5 September 2019 (22 days) by euthanizing fish with 200 mg/l buffered MS-222. Fish were not fed for 24 hours prior to sampling to ensure the digestive tract would be empty for energy reserve analyses. We measured total length, fork length and total mass and then dissected juveniles for further analyses (*n* = 397). A transverse cut was made directly behind the end of the operculum to separate the head from trunk (including all internal organs) of the fish. Trunks were reweighed to obtain wet weight prior to freezing. We placed all samples directly on dry ice and then stored them in −20°C freezers.

### Analysis of growth rate and energy reserves

Juvenile *A. aestivalis* are relatively intolerant of handling, and so we did not individually tag or mark individuals. Thus, we calculated direct measurements of specific growth rate at the tank level from Day 7 to the final day of each experiment using equation 1, where *M* represents mass, *f* represents final and *i* represents initial:.

(1) \begin{equation*} Specific\ growth\ rate=\frac{\ln ({M}_f)-\ln \left({M}_i\right)\ }{days}\times 100 \end{equation*}

We measured energy reserves in a subset of experimental fish (*n* = 170) using gravimetric methods described in Schultz and Conover (1997) and Guo *et al.* (2021) on whole trunk samples. Briefly, trunk samples were weighed in medium-porosity Alundum® extraction thimbles and then dried thimbles for at least 24 hours in a drying oven (60°C) to achieve a constant dry weight. Cooled samples and three dried, lipid-extracted juvenile river herring (lean control) were weighed to obtain a trunk dry mass. We placed the thimbles (including controls) in a custom-made, high-throughput Soxhlet apparatus (Schultz and Conover, 1997). Samples cycled through soaking in clean petroleum ether (extracts metabolically accessible lipids from tissues) for ~15 minutes followed by solvent flushing for 200 minutes, after which we returned thimbles to the drying oven (60°C) for at least 24 hours. Samples and controls were weighed to measure lean mass content. Next, dried ash controls (previously ashed) were weighed and placed with samples in a muffle furnace for 4 hours at 550°C. Samples were weighed directly from the oven (60°C); this ash mass represents non-metabolizable parts of the fish (e.g. bones). We calculated fat content (mg) as the total dried mass − lean mass. We calculated lean content (mg) as the lean mass − ash mass. Ash is assumed to have no energetic value to the fish.

We used methods described in Peig and Green (2009) to scale the fat and lean content of all juveniles to a common length to account for individual differences in final length. Briefly, the scaling power equation was used to model the relationship of energy reserve mass with total length. We utilized the standardized major axis (SMA) method in the *smatr* (Warton *et al.*, 2012) R package to estimate the scaling exponent for each component. Peig and Green (2009) proposed the use of SMA as the most appropriate technique for scaling mass components to account for variability in both the x- (length) and y- (mass) axes, while estimating the scaling exponent of interdependent variables. We used the power equation described in Peig and Green (2009) to calculate individual scaled fat mass-at-length and lean mass-at-length (${\hat{M}}_i$, also referred to as scaled mass index), where ${b}_{SMA}$ is the estimated scaling exponent and ${L}_o$ is the average length of all juveniles (equation 2).

(2) \begin{equation*} \hat{M}i={M}_i{\left[\frac{L_o}{L_i}\right]}^{b_{SMA}} \end{equation*}

To test whether a single value for ${b}_{SMA}$ was appropriate across experiments, we tested for any differences in estimated slopes between log-transformed total length and energy reserve mass for juveniles in each experiment. Differences in scaling exponents were identified for fat mass-at-length; thus after scaling all individuals with a single regression, we conducted all analyses for mass and energy reserve measures separately by experiment. Other studies have used similar approaches with least square regression (Ankney and Afton, 1988; Schultz and Conover, 1997). Literature values (Brett and Groves, 1979) were used to determine total caloric content (kCal) and energy densities (kCal/g) of collected trunk samples (equations 3 and 4).

(3) \begin{align*} Energy\ content&=\left({M}_{fat}\times 9.45\frac{kCal}{g}\right)\notag\\&\quad+\left({M}_{lean}\times 4.8\frac{kCal}{g}\right) \end{align*}

(4) \begin{equation*} Energy\ density=\frac{energy\ content}{M_{dry}} \end{equation*}

### Statistical methods

We conducted analyses in R version 3.5.3 (R Core Team, 2019) using packages *EnvStats* (Millard, 2013)*, survival* (Therneau, 2015) and *emmeans* (Lenth, 2020)*.* Prior to analyses, violations of the assumptions for each statistical test were checked. Generalized linear models (gamma distribution, log link function) were used to test for differences in mass at the beginning of each experimental round. In preparation for log-rank tests of survival probabilities, individuals that survived until sampling were assigned a status code of ‘1’ while fish that died during the experiment were assigned a status code of ‘2’, and their time to death was recorded in days. We used the Kaplan–Meier method to estimate survival probabilities over the course of each experiment and then used the *survminer* package (Kassambara *et al.*, 2021) to create Kaplan–Meier plots. Within experiments, we used nonparametric log-rank tests to analyse survival across temperatures. Multiple linear regression was used to test for differences in specific growth rate across temperatures and between experiments. To determine the appropriateness of analysing energy reserve results of both experiments in a single model, we tested for differences in linear relationships of log-transformed fat and lean mass-at-length with total length across experiments. Upon detecting differences in slopes for fat mass-at-length between experiments, analyses of individual measures were conducted separately for each experiment. We used generalized mixed effect models ‘GLMM’ (gamma distribution, log link function) to test the effects of temperature (fixed) and tank (random) on individual final mass, fat mass-at-length, lean mass-at-length and energy density within each experiment. Trunk dry mass was used as a covariate in models of fat mass-at-length and lean mass-at-length to determine whether there are intrinsic differences in energy reserves based on mass. We used total length instead of dry mass as a covariate in the energy density model, as energy density was calculated using dry trunk mass. In some cases, including tank as a random effect led to singular fitting, meaning the variance of some linear combinations of fixed effects in the fitted model were near zero. This result indicated that the random effect model structure was too complex to describe the dataset, and thus these models were rerun with only fixed effects and covariates.

## Results

**Figure 1:**
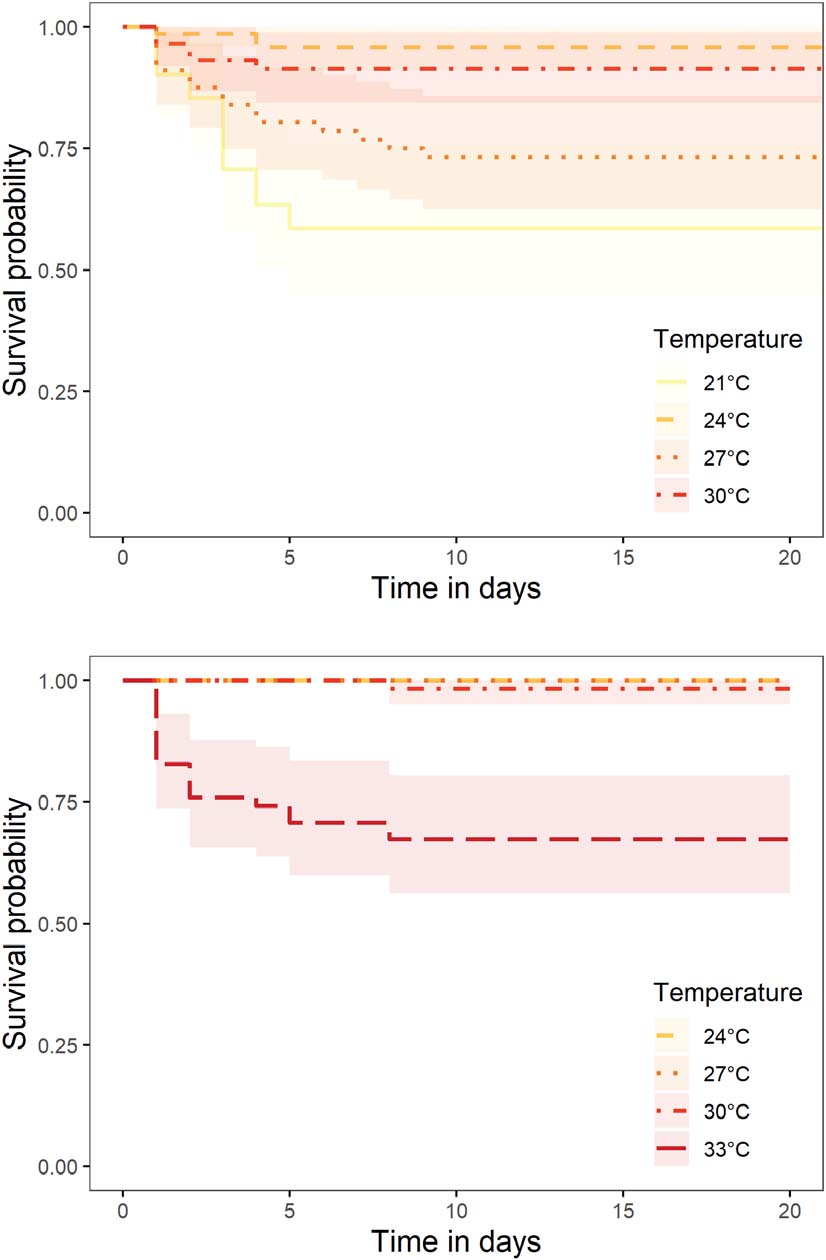
Kaplan–Meier plot of survival probabilities by temperature treatment in E1 (top) and E2 (bottom) for juvenile blueback herring (*A. aestivalis*) (E1, *n* = 41–71; E2, *n* = 56–59).

**Figure 2:**
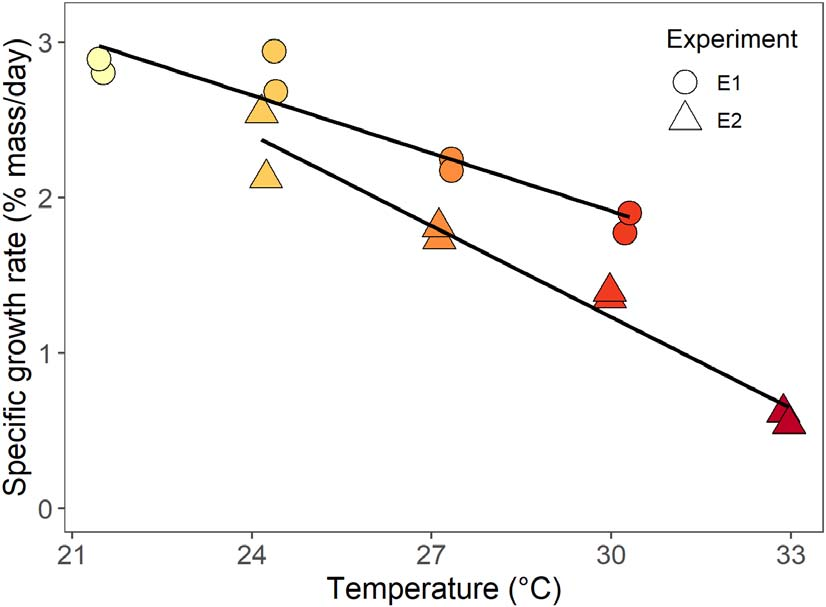
Specific growth rates (% mass/day) of juvenile blueback herring (*A. aestivalis*) at temperatures ranging from 21°C to 33°C in E1 (circles) and E2 (triangles) experiments (*n* = 2).

We examined the effects of temperature on juvenile survival and specific growth rates across experimental rounds. Most mortalities occurred within the first week of the experiment (Fig. 1). Survival differed among temperatures in E1 (log-rank test; χ^2^ = 32.2, df = 3, *P* < 0.001) and E2 (log-rank test; χ^2^ = 61.2, df = 3, *P* < 0.001). In E1, survival was maximized at 96% at 24°C and reached as low as 59% at 21°C. In E2, survival was near 100% for all temperatures except 33°C, where survival dropped to 67%. Overall, we observed lower juvenile survival in all E1 treatments compared with E2 treatments. E2 juveniles exhibited slower specific growth rates compared with E1 fish (multiple linear regression, *P* = 0.002; Fig. 2; Supplementary Table 2). Growth reached a maximum of 2.8% mass day^−1^ at 21°C and 24°C in E1 and a maximum of 2.3% at 24°C in E2; both experiments showed a steady decline in growth (slope, −0.16) as experimental temperature increased (*P* < 0.001). While growth rate was lowest at 33°C, juveniles still exhibited a positive growth rate of 0.6% biomass day^−1^.

We compared scaling exponents for fat mass-at-length and lean mass-at-length among experiments (i.e. testing for significant interactions between experiment and length; Table 1). The scaling exponent for fat mass-by-length relationships was almost 50% lower in E2 compared with E1 (likelihood-ratio test, *P* < 0.001; Table 1). There was no difference in scaling exponents between experiments for lean mass-by-length (*P* = 0.413).

**Table 1:** Estimated slopes and 95% confidence intervals of ln(fat mass) and ln(lean mass) regressed against ln(total length) for juvenile blueback herring (*A. aestivalis*), estimated using the SMA method (E1 *n* = 7–13; E2 *n* = 7–12)

	Combined slope	E1	E2	Experiment comparison		
	Estimate	Estimate	Estimate	Likelihood ratio	df	*P*
Fat × length	6.27 (6.02–6.54)	7.40 (6.43–8.53)	4.23 (3.62–4.95)	25.8	1	< 0.001
Lean × length	3.61 (3.53–3.70)	3.48 (3.15–3.85)	3.31 (3.07–3.56)	0.7	1	0.413

**Figure 3:**
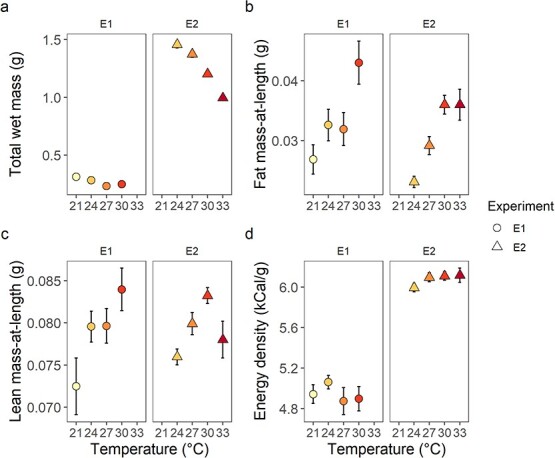
Temperature effects on juvenile blueback herring (*A. aestivalis*) (a) total wet mass (mean ± SE, *n* = 11–37), (b) fat mass-at-length (mean ± SE, *n* = 7–13), (c) lean mass-at-length (mean ± SE, *n* = 7–13) and (d) energy density (mean ± SE, *n* = 7–13) in each experiment. ^*^P < 0.05

We compared juvenile mass on the final day of the experiment among temperatures and examined the influence of temperature and size on fat mass-at-length, lean mass-at-length and energy density in each experiment (Fig. 3). In both rounds, wet mass decreased at temperatures ≥27°C (GLM, all *P* ≤ 0.038; Supplementary Table 3). Fish at the highest temperatures (30°C in E1, 33°C in E2) were on average 20.2% (GLM, *P* = 0.002) and 31.5% (*P* < 0.001) smaller, respectively, compared with fish maintained at the acclimation temperature. E2 juveniles were on average triple the mass of E1 juveniles (Fig. 3a). Size (mass or total length) had no effect on fat mass-at-length (GLMM; Supplementary Table 4; E1, *P* = 0.114; E2, *P* = 0.304), lean mass-at-length (GLMM; Supplementary Table 4; E1, *P* = 0.741; E2, *P* = 0.898) or energy density (GLM; Supplementary Table 5; E1, *P* = 0.215; E2, *P* = 0.930) in either experiment. E1 juveniles contained 64% more fat mass-at-length at 30°C compared with 21°C (*P* < 0.001), whereas E2 juveniles had 24–56% higher fat mass-at-length at higher temperatures compared with 24°C (all *P* < 0.050; Fig. 3b). At 30°C, lean mass-at-length increased 15% in E1 juveniles (*P* = 0.007) and increased 9% in E2 juveniles (*P* = 0.011) compared with juveniles at 21°C in E1 and 24°C in E2 (Fig. 3c). Lean mass-at-length was not different at any other temperature (all *P* ≥ 0.059). There was no difference in energy density with increasing temperatures in either experiment (all *P* ≥ 0.166; Fig. 3d). E2 juveniles were ~1 kCal g^−1^ more energy dense than E1 juveniles.

## Discussion

In this study, we examined the influence of temperature on juvenile blueback herring survival, growth and energy reserves when accounting for size. We found that increasing temperature affects juvenile growth rates and energy reserves, but also identified differences in survival, growth rate and fat mass-at-length between experiments, which suggest possible size-based differences in energy allocation.

We found some similarities in the impacts of temperature on growth and energy reserves across experiments. Temperatures ≥27°C led to slower growth rates and smaller juveniles. As temperatures rise, fish increase their routine metabolic rate to maintain normal cellular functions and repair damaged tissues to survive (Bernreuther *et al.*, 2013; Fry and Hart, 1948). Somatic growth is costly and occurs when energy intake is in excess of energetic needs, therefore supraoptimal temperatures typically limit energy available for somatic growth (Brett and Groves, 1979; Jobling, 1985). Approximately 40% of temperature measurements in three Connecticut River nursery coves (July and August 2019) were ≥27°C (Supplementary Fig. 1a), indicating that unless juveniles increase consumption rates or evade high temperatures, juveniles in this system may have depressed growth rates during the warmest months of freshwater residence. However, juveniles are unlikely to cease growth as temperatures ≥30°C were virtually never observed. Contrary to our expectations, fat and lean mass-at-length increased at 30°C compared with lower temperatures in both experiments, indicating growth in length is more limited by temperature than growth in fat and lean mass. Decreases in size typically correlate with a decrease in relative energy reserves (Schultz and Conover, 1997; Shuter and Post, 1990). Juveniles may have more constrained energy allocation to lean and fat reserves to maintain a minimum level of metabolizable energy reserves for survival (Wilkins, 1967), and indeed we observed no change in energy density across temperatures. Growth may be more plastic or less critical than maintaining a minimum level of energy reserves, since growth rates declined at lower temperatures than when scaled energy reserves increased. Thus, growth may be a more sensitive measure of temperature effects than either energy density or fat/lean content.

We also observed differences in juvenile responses to temperature between experiments. The lowest and highest temperatures used (21°C and 33°C) yielded the lowest survival. The high temperature of 33°C is virtually never observed in Connecticut River nursery habitats (Supplementary Fig. 1a) and some juveniles were unable to physiologically tolerate it. It is more unclear why 21°C elicited higher mortality, especially when surviving individuals maintained maximal growth rates. However *Saprolegnia sp*., a common pathogenic oomycete known to prefer colder temperatures, was observed more frequently on morbid or dead juveniles in 21°C tanks (Graham, 1956). Matching our predictions, lower overall survival among E1 juveniles compared with E2 may result from increased sensitivity due to their younger age, smaller size and/or individual or family-level variation. In fishes, larger juveniles are typically more resilient to suboptimal environmental conditions due to greater energetic reserves (Schultz and Conover, 1997; Sogard, 1997). E2 juveniles also maintained lower growth rates than E1 juveniles, possibly due to their larger size. In fishes consuming a maximum ration, larger fish allocate more energy to respiration and production of tissues and allocate less energy to growth (Brett and Groves, 1979; Smith, 1973). The final main difference between experiments was in the effects of size and temperature on fat mass-at-length reserves. E2 juveniles demonstrated a shallower positive slope than E1 juveniles for fat mass-at-length with increasing length, possibly resulting from an endogenous size-based shift in fat reserve accretion. E2 juveniles also maintained higher fat mass-at-length at all temperatures >24°C, while E1 juveniles only increased their fat mass-at-length at 30°C. Allometry of energetic composition can vary with body size and affects juvenile tolerance of physiological disturbances (Parker and Vanstone, 1966; Schultz and Conover, 1999). For instance, Schultz and Conover (1999) demonstrated size-mediated overwintering mortality wherein smaller Atlantic silversides (*Menidia menidia*) depleted their energy reserves at a faster rate and showed lower survival than larger silversides at 4°C and 8°C. Thus, the timing of starvation events or supraoptimal thermal regimes will influence juvenile fat reserves and fitness differently depending on juvenile size. Overall differences among experiments in juvenile survival and fat mass-at-length also may not necessarily arise from observed differences in juvenile size, but derive from genetic differences among cohorts, survivorship bias (i.e. juveniles surviving in nursery habitats in August may be more fit) or differences in thermal history.

Generally, our findings of high relative survival up to 33°C and a negative relationship of temperature and growth are not reflected in field studies on juvenile blueback herring. Juvenile abundance and densities can have positive, negative or neutral relationships with temperature (Devine *et al.*, 2021; Kosa and Mather, 2001; Tommasi *et al.*, 2015). Experimental design, differences in growth metrics (otoliths versus repeated mass measurements), behavioural thermoregulation (ability to choose thermal environment) or population-level differences in growth may explain differences in our findings. We fed experimental fish a consistent ration, whereas fish in natural systems can alter behaviour to find more food and generally experience more variable food availability. Field measures of juvenile growth rates have mainly suggested positive relationships between temperatures and growth (Alexander *et al.*, 2020; Dixon, 1996; Jessop, 1994; Tuckey, 2009). These relationships were identified in cooler systems or years (mean summer temperatures, 20.4–23.4°C), which could reflect the lower half of the thermal growth curve (Dixon, 1996; Jessop, 1994). There were also cases where high growth rates occurred during the coolest seasonal temperatures (Tuckey, 2009). Additionally, the use of degree days (Alexander *et al.*, 2020) assumes a linear relationship between degree days and growth, which is unlikely across the temperature range (7–31°C) and time period (April–September) examined. Our experimental approach allows us to clearly identify the sole influence on temperature on juvenile blueback herring physiology, but may overestimate field survival and growth rates in the absence of additional biotic and abiotic factors that further tax energetic reserves. Larval blueback herring may be more sensitive to high temperatures, as studies show significant increases in mortality with exposures to temperatures ≤26°C (Schubel *et al.*, 1977) and increasing mortality rates co-occurring with rising seasonal temperatures (Overton *et al.*, 2012).

While no data exist from field or experimental studies on energetic reserves and their importance for juvenile blueback herring, we expect a minimum level of energy reserves are critical for emigration and overwintering success as has been observed in other species. We observed two size classes of juvenile blueback herring similarly increase fat and lean mass-at-length to maintain a similar energy density, while lowering growth rates at high temperatures, up to a limit of 33°C. Energetic trade-offs are to be expected when temperatures become supraoptimal, resulting in more limited ‘excess’ energy available for routine metabolic costs (e.g. cellular maintenance), somatic growth and activity (Calow, 1984, 1985). In natural systems, this apparent switch in energy allocation from structural growth to maintaining an increased basal metabolic rate and energy reserves is likely influenced by additional ecological factors, including predator evasion, hypoxia and changing prey availability. If a minimum level of metabolizable energy reserves is required for survival or energetically costly activities, including emigration, preserving energy reserves through plasticity in growth rates and energy reserves may be critical (Adams *et al.*, 1985; Wilkins, 1967).

We observed a possible thermal upper limit of energy reserve plasticity at 33°C, when juveniles no longer linearly increased fat mass-at-length, and there was a decline in lean-mass-at-length. The constraint for increasing energy reserves at temperatures >30°C may result from increasingly limited energy availability, which allows for some fat reserves to be maintained, but not lean reserves. Lipid synthesis is relatively inexpensive energetically (15–25 mmol ATP g^−1^) compared with protein synthesis (70–100 mmol ATP g^−1^) (Buttery, 1973; Grisolia and Kennedy, 1966); thus juveniles may maintain fat synthesis and deposition over protein synthesis under extreme energetic constraints. Juvenile lean mass-at-length varied by ~15%, whereas fat mass-at-length varied by ~50%, supporting observations in many species that juvenile fishes are less plastic in allocations of lean mass-at-length across broad ranges of temperature (Brett *et al.*, 1969; Shearer, 1994). While high temperatures were required to alter fat and lean mass-at-length in this study, endogenous or exogenous factors that further limit available energy may result in increased sensitivity of energy reserves at lower temperatures (Matzelle *et al.*, 2015). Further research is required to understand juvenile blueback herring energy reserve dynamics in natural systems, with regards to temperature and other ecological factors.

Freshwater thermal regimes may partially determine juvenile blueback herring size distributions and thus population success. Blueback herring juvenile nursery habitat across eastern USA regularly reach mean summer temperatures ranging from 21°C to 30°C (Devine *et al.*, 2021; Kosa and Mather, 2001; Overton *et al.*, 2012; Tuckey, 2009), and may exceed 30°C in cases of power plant effluent or drought conditions (Supplementary Fig. 1). Temperature, when considered in conjunction with other nursery habitat factors such as dissolved organic carbon (DOC) and flow rates, accounts for variation in juvenile Alosa spp. densities and emigration success (Devine *et al.*, 2021; Tommasi *et al.*, 2015). Suitable, oxygenated thermal habitat occurs over a subset of the total physical freshwater habitat, and its extent changes over the period of Alosa spp. freshwater residency through shifts in the thermocline mediated by DOC levels (Devine *et al.*, 2021). If supraoptimal growing temperatures persist and juvenile blueback herring are unable to move to thermal refugia, they may be growth-limited and reach smaller sizes. Blueback herring appear to have a threshold size for emigration (Iafrate and Oliveira, 2008) and initiate emigration in response to low prey availability, lunar cycle and/or discharge (Kosa and Mather, 2001; Yako *et al.*, 2002). Delays in growth could delay emigration and expose juveniles to suboptimal temperatures or food conditions in nursery habitats. Furthermore, smaller juveniles may be more susceptible to size-mediated predation, environmental stressors and reduced performance (Juanes *et al.*, 1993; Sogard, 1997). Juvenile blueback herring have been observed to reside in diverse freshwater and estuarine habitats, even beyond their first year (Limburg and Turner, 2016; Payne Wynne *et al.*, 2015; Turner and Limburg, 2016), and movement among suitable habitats prior to emigration may help juveniles increase growth or energy reserves. If juveniles are unable to move between habitats during exposure to supraoptimal thermal conditions due to barriers like dams, dry outflows, hypoxic zones or predation risk, their resulting smaller size may lead to lower fitness and lack of long-term population stability or recovery.

There remain many challenges and knowledge gaps to implementing climate-ready management of blueback herring populations. The present results indicate that mid-range juvenile blueback herring appear to be well adapted for most local thermal regimes. However, climate model projections estimate that air temperatures in the lower Connecticut watershed could be >2°C warmer in 2050–2074 relative to 1981–2010, likely affecting water temperature and thus the physiology of mid-range juvenile blueback herring (Alder and Hostetler, 2013). It is less clear how southern populations are faring in current thermal regimes. While southern nursery habitats exceed 27°C more frequently in July and August (Supplementary Fig. 1b–d), differences in local adaptation, adult spawning phenology and/or length of growing season could offset depressed juvenile growth rates predicted by our study (Legett *et al.*, 2021; Lombardo *et al.*, 2019; Tommasi *et al.*, 2015). Hypothetically, at the southern range edge, populations are living closer to thermal limits and could be more sensitive to variation in environmental factors (Hochachka and Somero, 2002; Neill *et al.*, 1994); because early life stages have limited capacity to evade poor environmental conditions, range-edge population success may be more strongly influenced by early life stage dynamics. Indeed, southern range contractions of blueback herring populations are ongoing, though a mechanistic link to climate change has not yet been made (Nye *et al.*, 2012). Replication of our study including southern populations of juvenile blueback herring could help clarify whether local thermal adaptation exists and if southern range contractions could be explained by decreased survival, growth or energy reserves in early life stages. As thermal regimes become warmer and more variable across the blueback herring range, we can expect changes in adult spawning phenology, zooplankton dynamics and other species interactions to act in antagonistic or synergistic ways with temperature and other habitat factors to affect juvenile physiology (Brodersen *et al.*, 2011; Legett *et al.*, 2021; Lombardo *et al.*, 2019). Thus, it could be beneficial to further investigate and incorporate the impacts of temperature and other ecological factors into management of blueback herring populations in order to improve the efficacy of recovery efforts of this species, thereby benefitting aquatic ecosystems that depend on plentiful, energy-rich prey items (Dias *et al.*, 2019; Walters *et al.*, 2009).

Temperature is an important factor influencing the physiology and thus ecology of fishes at all life stages. By affecting size and energy allocation, temperature determines what energy is available for critical metabolic processes and activities. The strength of selection for larger size is approximately five times greater for fishes than for terrestrial taxa, demonstrating how damaging temperature-limited growth can be for the sustainability of fish populations (Perez and Munch, 2010). Understanding how climate change will influence limited energy budgets is particularly important for anadromous fishes, many of which are imperilled and are challenged by energy-intensive changes in physiology or behaviour during multiple life stages (Limburg and Waldman, 2009). Each species may have diverse energy allocation strategies during different life stages, seasons or locations (Pedersen and Hislop, 2001; Rätz and Lloret, 2003; Schultz and Conover, 1999). Identifying species-specific life stages, time periods and populations that are most sensitive to negative physiological effects of temperature can help conservation efforts become more targeted and effective. To accomplish this, management efforts could aim to clarify and mitigate the negative impacts of temperature by prioritizing restoration and management of cool-water freshwater systems to improve population success through increased fecundity, survival and ecophysiological resilience (Brosset *et al.*, 2016; Rätz and Lloret, 2003; Sogard, 1997).

## Funding

This work was supported by the US Geological Survey Science Support Program and the University of Massachusetts Amherst Department of Environmental Conservation.

## Contributions

L.G. contributed to experimental design, funding, data generation, data analysis and manuscript preparation. A.J. and E.S. contributed to experimental design, data analysis and manuscript preparation. S. M. contributed to experimental design, funding, data generation, data analysis and manuscript preparation.

## Data Availability Statement

The original data underlying this article will be shared on reasonable request to the corresponding author. Some of the temperature measurement data underlying the supplemental material in this article were provided by Dr Troy Tuckey and the North Carolina Division of Marine Fisheries by permission. This content is not covered by the terms of the Creative Commons licence of this publication. For permission to reuse, please contact the rights holder. Presentation of North Carolina Division of Marine Fisheries temperature data are non-Division use of their data. US Geological Survey gage data can be accessed at https://waterdata.usgs.gov/nwis.

## Supplementary Material

Web_Material_coac022
