# Supplementary material for: Identification of supraoptimal temperatures in juvenile blueback herring (Alosa aestivalis) using survival, growth rate and scaled energy reserves
Source: Conserv Physiol. 2022 Apr 20;10(1):coac022. doi: 10.1093/conphys/coac022 (PMC9040283; doi:10.1093/conphys/coac022)
Supplement: Web_Material_coac022 [file web_material_coac022.docx]

**Supplemental Materials:** Identification of supraoptimal temperatures in juvenile blueback herring (*Alosa aestivalis*) using survival, growth rate and scaled energy reserves

*Any use of trade, firm, or product names is for descriptive purposes only and does not imply endorsement by the U.S. Government.*

Supplementary Table 1. Comparison of average juvenile blueback herring (Alosa aestivalis) mass between experimental tanks on day -2 using generalized linear models with a gamma distribution and log link function (E1 n = 26 - 40, E2 n = 28 - 36).

|  | **E1** | | | |  | **E2** | | | |
| --- | --- | --- | --- | --- | --- | --- | --- | --- | --- |
|  | *Estimate* | *SE* | *t value* | *Pr (>\|t\|)* |  | *Estimate* | *SE* | *t value* | *Pr (>\|t\|)* |
| Intercept | 4.929 | 0.063 | 78.04 | < 0.001 |  | 6.797 | 0.041 | 164.29 | < 0.001 |
| Tank 2 | 0.062 | 0.083 | 0.75 | 0.453 |  | -0.105 | 0.059 | -1.78 | 0.076 |
| Tank 3 | 0.031 | 0.083 | 0.37 | 0.709 |  | -0.038 | 0.058 | -0.66 | 0.508 |
| Tank 4 | 0.126 | 0.084 | 1.49 | 0.138 |  | 0.039 | 0.056 | 0.70 | 0.488 |
| Tank 5 | -0.028 | 0.092 | -0.31 | 0.759 |  | 0.031 | 0.058 | 0.53 | 0.595 |
| Tank 6 | -0.088 | 0.085 | -1.03 | 0.303 |  | -0.004 | 0.060 | 0.06 | 0.953 |
| Tank 7 | -0.090 | 0.087 | -1.04 | 0.301 |  | 0.003 | 0.059 | 0.06 | 0.956 |
| Tank 8 | -0.109 | 0.087 | -1.25 | 0.214 |  | 0.010 | 0.058 | 0.18 | 0.859 |

Supplementary Table 2. Multiple linear regression modelling tank specific growth rate (% mass/day) of juvenile blueback herring (Alosa aestivalis) across experimental rounds (E1 or E2) and temperatures (E1: 21°C, 24°C, 27°C, 30°C; E2: 24°C, 27°C, 30°C, 33°C) (n = 2).

|  | **Specific growth rate (% mass/day)** | | | |
| --- | --- | --- | --- | --- |
|  | *Estimate* | *SE* | *t value* | *Pr (>\|t\|)* |
| Intercept | 6.56 | 0.40 | 16.22 | < 0.001 |
| Temperature (°C) | -0.16 | 0.02 | -10.37 | < 0.001 |
| Round | -0.48 | 0.11 | -4.47 | <0.001 |

Supplementary Table 3. Generalized linear models (gamma distribution and log link function) testing the effects of temperature on juvenile blueback herring (Alosa aestivalis) final mass (g) within experimental rounds (E1 n = 11 - 37; E2 n = 19 - 31)

|  | **E1** | | | |  |  | **E2** | | | |
| --- | --- | --- | --- | --- | --- | --- | --- | --- | --- | --- |
|  | *Estimate* | *SE* | *t value* | *Pr (>\|t\|)* |  |  | *Estimate* | *SE* | *t value* | *Pr (>\|t\|)* |
| Intercept | -1.166 | 0.059 | -19.61 | < 0.001 |  | Intercept | 0.377 | 0.020 | 18.74 | < 0.001 |
| 24°C | -0.101 | 0.069 | -1.46 | 0.147 |  | 27°C | -0.059 | 0.028 | -2.09 | 0.038 |
| 27°C | -0.299 | 0.075 | -3.99 | < 0.001 |  | 30°C | -0.192 | 0.028 | -6.92 | < 0.001 |
| 30°C | -0.230 | 0.072 | -3.20 | 0.002 |  | 33°C | -0.381 | 0.031 | -12.15 | < 0.001 |

Supplementary Table 4. Generalized linear mixed effect models (gamma distribution and log link function) testing the effects of temperature (fixed), tank (random), and dry trunk mass (covariate) on juvenile blueback herring (Alosa aestivalis) fat mass-at-length (g) and lean mass-at-length (g) within each experimental round (E1 n = 7 - 13; E2 n = 7 - 12).

|  | **E1** | | | | | | | | |
| --- | --- | --- | --- | --- | --- | --- | --- | --- | --- |
|  | Fat mass-at-length | | | |  | Lean mass-at-length | | | |
|  | *Estimate* | *SE* | *t value* | *Pr (>\|t\|)* |  | *Estimate* | *SE* | *t value* | *Pr (>\|t\|)* |
| Intercept | -3.905 | 0.207 | -18.84 | < 0.001 |  | -2.647 | 0.075 | -35.17 | < 0.001 |
| Dry mass (g) | 5.283 | 3.339 | 1.58 | 0.114 |  | 0.395 | 1.194 | 0.33 | 0.741 |
| 24°C | 0.201 | 0.136 | 1.48 | 0.140 |  | 0.095 | 0.050 | 1.89 | 0.059 |
| 27°C | 0.247 | 0.148 | 1.68 | 0.093 |  | 0.100 | 0.054 | 0.19 | 0.062 |
| 30°C | 0.543 | 0.153 | 3.54 | < 0.001 |  | 0.153 | 0.056 | 2.72 | 0.007 |
|  |  |  |  |  |  |  |  |  |  |
| Random effect | Variance | SD |  |  |  | Random effect | Variance | SD |  |
| Tank | 0.001 | 0.032 |  |  |  | Tank | < 0.001 | 0.012 |  |
| Residual | 0.162 | 0.402 |  |  |  | Residual | 0.020 | 0.141 |  |
|  |  |  |  |  |  |  |  |  |  |
|  | **E2** | | | | | | | | |
|  | Fat mass-at-length | | | |  | Lean mass-at-length | | | |
|  | *Estimate* | *SE* | *t value* | *Pr (>\|t\|)* |  | *Estimate* | *SE* | *t value* | *Pr (>\|t\|)* |
| Intercept | -3.603 | 0.174 | -20.74 | < 0.001 |  | -2.584 | 0.057 | -45.11 | < 0.001 |
| Dry mass (g) | -0.481 | 0.469 | -1.03 | 0.304 |  | 0.019 | 0.149 | 0.13 | 0.898 |
| 27°C | 0.221 | 0.090 | 2.44 | 0.015 |  | 0.049 | 0.036 | 1.28 | 0.166 |
| 30°C | 0.413 | 0.092 | 4.47 | < 0.001 |  | 0.093 | 0.036 | 2.54 | 0.011 |
| 33°C | 0.385 | 0.104 | 3.72 | < 0.001 |  | 0.022 | 0.040 | 0.56 | 0.577 |
|  |  |  |  |  |  |  |  |  |  |
| Random effect | Variance | SD |  |  |  | Random effect | Variance | SD |  |
| Tank | 0.001 | 0.034 |  |  |  | Tank | < 0.001 | 0.017 |  |
| Residual | 0.052 | 0.229 |  |  |  | Residual | 0.006 | 0.076 |  |

Supplementary Table 5. Generalized linear models (gamma distribution and log link function) testing the effects of temperature and total length (covariate) on juvenile blueback herring (Alosa aestivalis) energy density (kCal/g) within experimental rounds (E1 n = 7 - 13; E2 n = 7 - 12)

|  | **E1** | | | |  |
| --- | --- | --- | --- | --- | --- |
|  | *Estimate* | *SE* | *t value* | *Pr (>\|t\|)* |  |
| Intercept | 1.444 | 0.141 | 10.25 | < 0.001 |  |
| Total length (mm) | 0.004 | 0.004 | 1.08 | 0.284 |  |
| 24°C | 0.028 | 0.030 | 0.93 | 0.356 |  |
| 27°C | 0.000 | 0.033 | 0.00 | 0.999 |  |
| 30°C | 0.010 | 0.037 | 0.27 | 0.792 |  |

|  | **E2** | | | |
| --- | --- | --- | --- | --- |
|  | *Estimate* | *SE* | *t value* | *Pr (>\|t\|)* |
| Intercept | 1.801 | 0.089 | 20.28 | < 0.001 |
| Total length (mm) | 0.000 | 0.002 | -0.10 | 0.919 |
| 27°C | 0.017 | 0.012 | 1.40 | 0.166 |
| 30°C | 0.018 | 0.014 | 1.33 | 0.187 |
| 33°C | 0.019 | 0.015 | 1.25 | 0.216 |


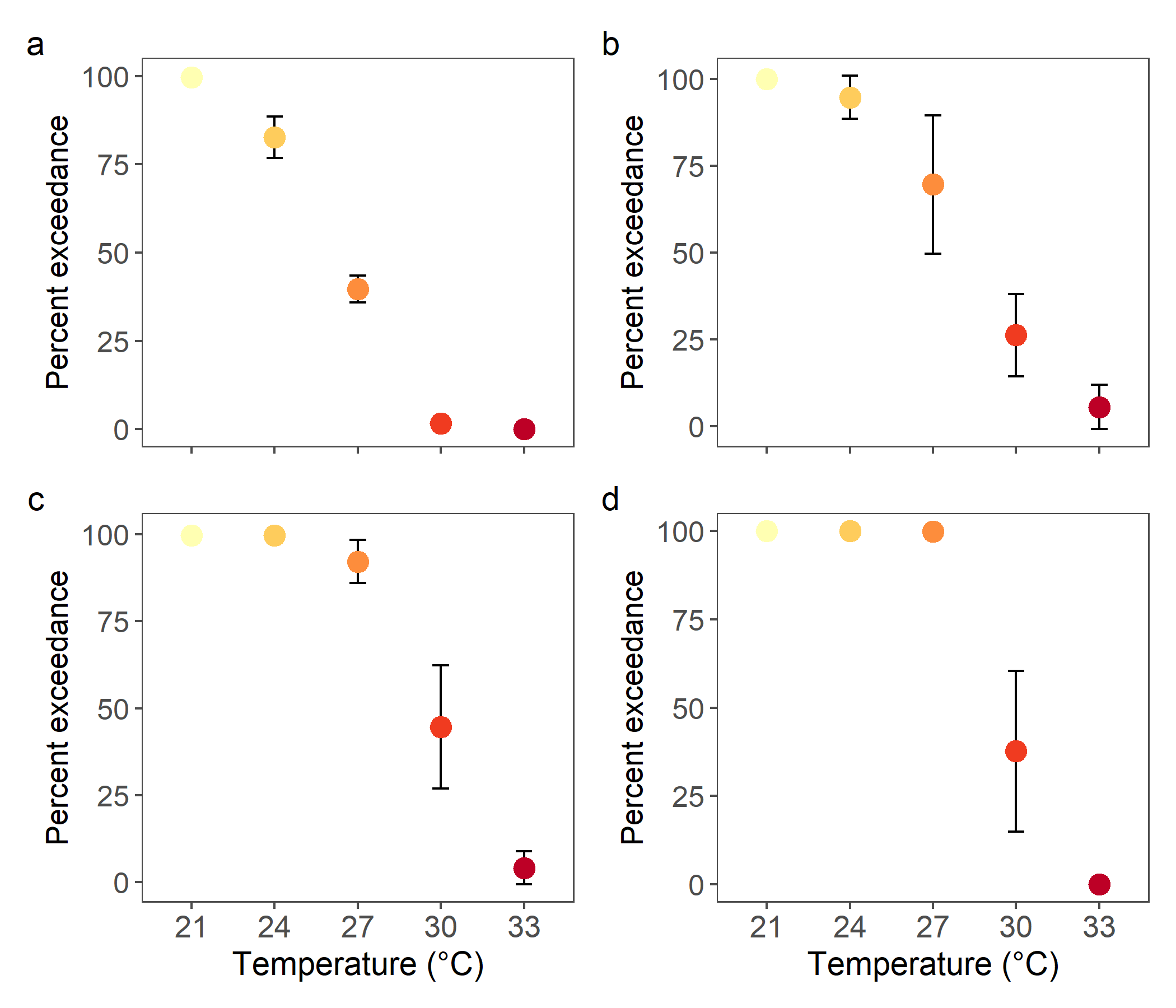


Supplementary Figure 1. Mean (± SD) percentage exceedance at a given temperature in July and August in juvenile blueback herring (Alosa aestivalis) nursery sites for a) three sites in the Connecticut River (Hamburg Cove, Salmon Cove, and Wethersfield Cove) in 2019 measured using HOBO loggers at 20 minute intervals, approximately 1 m deep in the water column, b) four sites in Virginia (Mattaponi, Pamunkey, James, Rappahannock) in 2005 – 2007 reported published in Tuckey 2009^1^, c) eleven sites in North Carolina (Harvey Point, Sandy Point, Batchelor Bay, Albemarle Sound Bridge, Yeopim Creek, Chowan River, Sheep's Landing, Tuscarora Beach, Arrowhead Beach, Scuppernong River, Colonial Beach) in 2010 – 2020 from monthly juvenile river herring surveys conducted by the NC Division of Marine Fisheries^2^, and d) three sites in the Saint Johns River (Astor, Buffalo Bluff, Lake George) in 2012 – 2015 obtained from USGS gage data.

**Notes:**

1. Virginia nursery site temperature measurements
   Tuckey T, Variability in Juvenile Growth, Mortality, Maturity, and Abundance of American Shad and Blueback Herring in Virginia, 2009, by permission of Troy Tuckey.
   This content is not covered by the terms of the Creative Commons licence of this publication. For permission to reuse, please contact the rights holder.
2. Presentation of NC Division of Marine Fisheries temperature data are non-Division use of their data.
